# Investigating intraoperative parathyroid hormone criteria for enhanced accuracy and surgical success in treating primary hyperparathyroidism: results from two tertiary referral centres

**DOI:** 10.1093/bjsopen/zraf055

**Published:** 2025-06-13

**Authors:** Eleonora Lori, Loredana De Pasquale, Alberto M Saibene, Luca Castellani, Daniele Pironi, Piergaspare Palumbo, Domenico Tripodi, Flavio Forte, Corrado De Vito, Gaetano Gallo, Salvatore Sorrenti

**Affiliations:** Department of Surgery, ‘Sapienza’ University of Rome, Rome, Italy; Thyroid and Parathyroid Surgery Service–Otolaryngology Unit, ASST Santi Paolo e Carlo, Department of Health Sciences, University of Milan, Milan, Italy; Otolaryngology Unit, ASST Santi Paolo e Carlo, Department of Health Sciences, University of Milan, Milan, Italy; Otolaryngology Unit, ASST Santi Paolo e Carlo, Department of Health Sciences, University of Milan, Milan, Italy; Department of Surgery, ‘Sapienza’ University of Rome, Rome, Italy; Department of Surgery, ‘Sapienza’ University of Rome, Rome, Italy; Department of Surgery, ‘Sapienza’ University of Rome, Rome, Italy; Department of Urology, M. G. Vannini Hospital, Rome, Italy; Department of Sciences, Roma Tre University, Rome, Italy; Department of Public Health and Infectious Diseases, ‘Sapienza’ University of Rome, Rome, Italy; Department of Surgery, ‘Sapienza’ University of Rome, Rome, Italy; Department of Surgery, ‘Sapienza’ University of Rome, Rome, Italy

## Abstract

**Background:**

Primary hyperparathyroidism is a common endocrine disorder necessitating surgical intervention for definitive treatment. Measurement of intraoperative parathyroid hormone (ioPTH) has revolutionized surgical management, but interpreting the results remains a subject of debate. This retrospective study, evaluated the efficacy of the Miami criterion (a > 50% decrease in ioPTH level 10 minutes after parathyroid removal) in predicting surgical success and whether meeting this criterion reduced persistence rates. It also investigated whether achieving a drop in ioPTH concentration to within the normal range, either alone or in combination with meeting the Miami criterion, led to improved outcomes.

**Methods:**

A retrospective analysis was conducted on patients with primary hyperparathyroidism who underwent surgery at two Italian tertiary referral centres. Univariate and multivariate analyses were used to identify predictors of persistence. The diagnostic performances of both ioPTH criteria were assessed, individually and in combination.

**Results:**

Data from 380 patients were analysed. Multivariate analysis confirmed the efficacy of both ioPTH criteria, showing a negative association between persistence and both a fall to within the normal range (*P* = 0.005) and a > 50% decrease in ioPTH level (*P* = 0.039). The > 50% reduction in ioPTH criterion demonstrated higher sensitivity (95.0%) and lower specificity (45.0%) than the normalization of ioPTH criterion (sensitivity 81.1% and specificity 60.0%). Combining the two criteria resulted in the highest sensitivity (97.5%) and an improved negative predictive value (55.0%) compared with each criterion alone, resulting in the highest accuracy.

**Conclusion:**

A fall in ioPTH level to within the normal range helps prevent persistence, whereas a drop of > 50% reduces the rate of unnecessary bilateral neck explorations. Combining the two criteria yields the best results in terms of predicting surgical outcome.

## Teaser

This study evaluated the efficacy of the Miami criterion in predicting surgical success and persistence, and whether achieving a decrease in intraoperative parathyroid hormone (ioPTH) level to within the normal range leads to improved outcomes. Combining the two criteria yielded the best results: when the decrease in ioPTH concentration is < 50% but falls to within the normal range, there is no increased risk of persistence.

## Introduction

Primary hyperparathyroidism is a relatively common endocrine disorder characterized by excessive secretion of parathyroid hormone (PTH), leading to abnormally high levels of calcium in the blood^1^. In approximately 85% of instances, a single adenoma causes primary hyperparathyroidism, whereas in multiple parathyroid glands are involved 15%, either in the form of hyperplasia or multiple adenomas^2^. Parathyroid carcinomas are rare, representing less than 1% of cases of primary hyperparathyroidism. The only definitive treatment for primary hyperparathyroidism is surgery^3^. However, determining the cause of the disease and the exact number of affected glands is currently not possible before surgery^4^.

The traditional approach to treating primary hyperparathyroidism involves bilateral neck exploration (BNE) to identify all macroscopically pathological glands and to remove hyperfunctioning parathyroid tissue^5,6^. Surgical challenges arise not only from the need to know how many glands are hyperfunctioning but also from the difficulty of locating the glands precisely due to considerable variability in their number and location^7^. Reintervention for persistence or recurrence, especially in the neck region, can be particularly challenging and is associated with a high risk of complications^8^. Therefore, to consider a patient cured, the surgeon must reasonably ensure the removal of all hyperfunctioning parathyroid tissue before concluding the surgical procedure.

The introduction of rapid monitoring of intraoperative parathyroid hormone (ioPTH)^9^ has allowed surgeons to assess whether the gland causing the primary hyperparathyroidism has been removed successfully, thereby reducing the risk of persistence. Over the years, minimally invasive parathyroidectomy (MIP) has become the most common minimally invasive technique. MIP entails surgically addressing only the gland with preoperative suspicious characteristics, minimizing surgical dissection and potential complications. More invasive procedures, such as BNE, are required only if indicated by intraoperative examinations^10^.

Various criteria for interpreting ioPTH levels have been developed over the years to determine the success of the surgical intervention^11^. Among the most widely adopted is the Miami criterion, defined as a decrease of > 50% between the PTH concentration at baseline (or immediately before excision) and 10 minutes (min) after excision, which has an estimated accuracy of approximately 97%^12,13^. The Miami criterion balances the need to avoid excessively prolonged operating times associated with the use of ioPTH measurement (in the context of requiring specialized equipment in the operating room to perform the test) and the importance of minimizing unnecessary BNE^13–15^.

The aim of the present study was to determine whether the Miami criterion is sufficient to predict no disease persistence without unnecessary BNE. In addition, the study investigated whether achieving a drop in PTH level to within the normal range (NR), alone or in combination with meeting the Miami criterion, results in better outcomes.

## Methods

### Study design

A retrospective review was undertaken using a prospectively maintained database of 540 consecutive patients diagnosed with hyperparathyroidism who underwent parathyroidectomy performed by high-volume endocrine surgeons at two Italian tertiary referral centres (Thyroid and Parathyroid Unit, Santi Paolo e Carlo Hospital, Milan; and Department of Surgical Science, Sapienza University of Rome, Rome) between 2001 and 2022.

All patients provided informed consent for inclusion in the institutional database, where information regarding their clinical data (diagnosis, hospitalization, and follow-up) and written informed consent before surgery was recorded meticulously. This study was conducted in accordance with the principles outlined in the Declaration of Helsinki and complied with the STROBE checklist (*Table S1*)^16^.

**Fig. 1:**
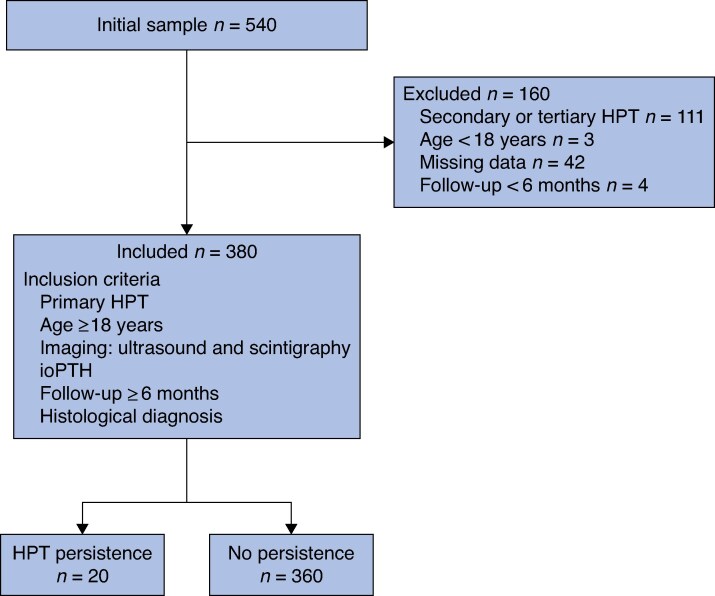
Flow chart showing inclusion and exclusion criteria HPT, hyperparathyroidism; ioPTH, intraoperative parathyroid hormone.

Only patients affected by primary hyperparathyroidism who were undergoing parathyroidectomy and for whom a minimum 6-month follow-up was available were included in the study. All patients included in the analysis were adults (aged ≥ 18 years) and all underwent preoperative ultrasonography and scintigraphy, ioPTH measurement (at induction and 10 min after parathyroid removal), and histological analysis of the removed glands. Patients diagnosed with secondary or tertiary hyperparathyroidism (111) were excluded from the study, as were patients with missing data that could not be retrieved from initial paper records (42), those aged < 18 years (2), and those with a follow-up duration of < 6 months (4) (*Fig. 1*).

The diagnosis of primary hyperparathyroidism relied on assessing blood concentrations of calcium, phosphorus, PTH, and 25-hydroxyvitamin D, as well as 24-hour urinary calcium. Patients with preoperative imaging suggestive of single-gland disease underwent MIP. If preoperative imaging was not suggestive of a single adenoma, or in the event of concurrent thyroidectomy, patients underwent parathyroidectomy with BNE. In both situations, an ioPTH assay was conducted. The decision to convert from MIP to BNE was based on the decline in ioPTH levels.

True-positives were defined as patients in whom the required decrease in ioPTH level was achieved and who did not experience a recurrence of the disease within 6 months after surgery. True-negatives were defined as patients in whom the ioPTH level did not decrease, either because of multiglandular disease or incomplete removal of pathological parathyroid tissue. Following the removal of pathological parathyroid tissue, ioPTH measurement was repeated, but disregarded. False-positives were defined as patients in whom the ioPTH concentration declined, despite all or part of the pathological tissue not being removed, as confirmed by an increase in PTH levels during the 6-month follow-up. False-negatives were defined as patients who the ioPTH criteria classified inaccurately as requiring removal of parathyroid tissue and who subsequently underwent unnecessary BNE, with no other glands excised and no persistence of the disease observed during the 6-month follow-up.

The ioPTH data were analysed retrospectively, using the drop in ioPTH level to within the NR as a reference. Patients were considered cured and the surgery concluded only when the ioPTH concentration returned to within the NR. Finally, the two ioPTH criteria were combined, and treatment was considered effective if patients exhibited a > 50% decrease in PTH concentration relative to baseline or if the PTH level fell to within the NR. Using these criteria, the surgery continued and BNE was performed only when the decrease in PTH level was < 50% and the ioPTH concentration at 10 min after parathyroid removal remained above the NR.

Patient data were evaluated, including: demographic data (age at surgery, sex); preoperative biochemical data (PTH, calcium, vitamin D, and urinary calcium levels); imaging results (neck ultrasound imaging or computed tomography and scintigraphy); ioPTH measurements (at baseline and 10 min after gland removal); results of histological examinations; gland weight (when available); and surgical outcomes (with a follow-up of at least 6 months). The NR for PTH was 8.7–79.6 pg/ml and that for serum calcium was 8.4–10.2 mg/dl. Persistent hyperparathyroidism was defined by the presence of raised PTH levels and hypercalcaemia immediately after or within 6 months of surgery, with reassessment of calcaemia, PTH, and vitamin D levels at the 6-month follow-up.

### Statistical analysis

Continuous variables are reported as mean(standard deviation) or median (range); categorical variables are reported as absolute and relative frequencies. The significance of differences between groups was evaluated using Student's *t* test for continuous variables and the χ^2^ test for categorical variables. A multivariable logistic regression model was built to identify predictors of persistence. Variables were included in the model based on the results of univariate analysis (*P* < 0.250) and on expert opinion. Multicollinearity was checked using a variance inflation factor of 5 as the threshold. The Hosmer–Lemeshow test was used to evaluate the goodness of fit of the model. Odds ratios and 95% confidence intervals were calculated. All analyses were performed using Stata^®^ version 17.0 (StataCorp, College Station, TX, USA). Two-sided *P* < 0.050 was considered statistically significant.

Sensitivity, specificity, positive predictive value (PPV), negative predictive value (NPV), and accuracy were evaluated for the three different ioPTH criteria. Confidence intervals were calculated using the Clopper–Pearson method.

A sensitivity analysis was performed to evaluate the influence of specific variables on the outcome of the multivariate analysis and the diagnostic performance of the criteria.

## Results

The clinical characteristics of the patients are summarized in *Table 1*. Of the 540 patients in the database, 380 were eligible for inclusion in the present study. Of these, 295 were women (77.6%) and 85 were men (22.4%), with a median age of 61.5 (range 20–85) years. All patients were followed up for a minimum of 6 months, with a median follow-up of 12 (range 1–45) months. For patients who experienced persistence, follow-up was considered concluded at the time persistence was diagnosed. MIP was performed in 251 patients (66.0%), and 129 patients (34.0%) underwent BNE, 68 of whom underwent concurrent thyroidectomy. In all, 353 of 380 patients (92.9%) achieved a decrease in PTH level of > 50%, but it returned to the NR in only 300 patients (78.9%). Histological analysis confirmed a single parathyroid adenoma in 324 patients (85.4%), consistent with rates reported in the literature^2^. Multiglandular disease was observed in 39 patients (10.3%), including 37 with hyperplasia.

**Table 1:** Clinical features of patients with primary hyperparathyroidism overall and according to the persistence of hyperparathyroidism

	Overall (*n* = 380)	No persistence (*n* = 360)	Persistence (*n* = 20)	P*
**Sex**				0.794†
Female	295 (77.6%)	279 (77.5%)	16 (80.0%)	
Male	85 (22.4%)	81 (22.5%)	4 (20.0%)	
Age at surgery (years), median (range)	61.50 (20–85)	61.50 (20–85)	62.00 (32–79)	0.860
Preoperative serum calcium (mg/dl), mean(s.d.)	11.89(1.38)	11.92(1.34)	11.314(1.90)	0.003
Preoperative PTH (pg/ml), mean(s.d.)	330.88(452.88)	334.89(460.55)	258.75(78.80)	0.361
**Ultrasound imaging**				0.490†
Positive	283 (74.5%)	271 (75.3%)	12 (60.0%)	
Negative	97 (25.5%)	89 (24.7%)	8 (40.0%)	
**Scintigraphy**				0.272†
Positive	270 (71.1%)	259 (71.9%)	10 (50.0%)	
Negative	110 (28.9%)	101 (28.1%)	10 (50.0%)	
Baseline PTH (pg/ml), mean(s.d.)	311.53(485.70)	315.00(495.24)	249.05(259.41)	0.938
ioPTH at 10 min (pg/ml), mean(s.d.)	57.03(68.37)	54.15(66.50)	108.86(81.83)	0.267
**Reduction in ioPTH > 50%**				< 0.001†
Yes	353 (92.9%)	342 (95.0%)	11 (55.0%)	
No	27 (7.1%)	18 (5.0%)	9 (45.0%)	
**Fall in ioPTH to within normal range**				< 0.001†
Yes	300 (78.9%)	292 (81.1%)	8 (40.0%)	
No	80 (21.1%)	68 (18.9%)	12 (60.0%)	
**Surgical approach**				0.284†
MIP	251 (66,0%)	240 (66.7%)	11 (55.0%)	
Open surgery	129 (34,0%)	120 (33.3%)	9 (45.0%)	
**Concurrent thyroidectomy**				0.040†
Yes	68 (17.9%)	61 (16.9%)	7 (35.0%)	
No	312 (82.1%)	299 (83.1%)	13 (65.0%)	
PTH on POD 1 (pg/ml), mean(s.d.)	34.37(38.87)	28.41(22.94)	141.55(86.19)	0.003
**Histological finding**				< 0.001†
Single adenoma	324 (85.4%)	316 (87.7%)	8 (40.0%)	
Double adenoma	2 (0.5%)	2 (0.6%)	0 (0.0%)	
Hyperplasia	37 (9.7%)	30 (8.3%)	7 (35.0%)	
Carcinoma	10 (2.6%)	10 (2.8%)	0 (0.0%)	
Inconclusive	7 (1.8%)	2 (0.6%)	5 (25.0%)	
Multiglandular disease	39 (10.3%)	34 (9.4%)	7 (35.0%)	< 0.001†
Single-gland disease	334 (87.9%)	326 (90.6%)	13 (65.0%)	
MEN1	4 (1.1%)	3 (0.8%)	1 (5.0%)	0.076†
Parathyroid weight (mg), mean (s.d.)	1874.60(4359.62)	1919.30(4423.57)	681.00(720.56)	0.176
Postoperative serum calcium (mg/dl), mean(s.d.)	9.62(1.21)	9.12(0.73)	11.237(1.67)	< 0.001
Postoperative PTH (pg/ml), mean(s.d.)	36.98(33.61)	24.53(14.77)	133.84(55.23)	< 0.001

s.d., Standard deviation; PTH, parathyroid hormone; ioPTH, intraoperative parathyroid hormone; MIP, minimally invasive parathyroidectomy; POD, postoperative day; MEN1, multiple endocrine neoplasia type 1. *Student's *t* test, except †χ^2^ test.

Four patients were diagnosed with multiple endocrine neoplasia type 1 (MEN1). Three of these four patients underwent subtotal parathyroidectomy, facilitated by the role of ioPTH measurement. One patient had two glands removed, with an adequate decrease in ioPTH concentration, but experienced disease recurrence during follow-up, with histological diagnosis of parathyroid hyperplasia. The diagnosis of MEN1 was made after surgery in three of the four patients. When a preoperative diagnosis of MEN1 was established, the preferred approach was open surgery with BNE.

In seven patients, no pathological parathyroid tissue was excised and a histological diagnosis was not possible. Among these patients, no parathyroid tissue was removed at all in two patients because no pathological gland could be identified in the neck, and the procedure was concluded without resolving the clinical condition. In the remaining five patients, a gland was removed based on its macroscopic appearance, but histological examination revealed it to be healthy. Among these seven patients, three had concordant preoperative imaging and four underwent concurrent thyroidectomy. Overall, only two patients underwent minimally invasive video-assisted parathyroidectomy, whereas five underwent BNE. Therefore, there were no data suggesting multiglandular disease, but rather an ectopic localization of the pathological gland. In five of these seven patients, the ioPTH level decreased sufficiently that the procedure was considered concluded; this decrease could possibly have been due to ischaemic damage to the parathyroid glands related to the neck exploration, with PTH levels subsequently increasing on the first day after surgery.

Overall, 360 patients were cured, whereas 20 (5.3%) had persistent disease. Of the 20 patients with persistent disease, 8 met both ioPTH criteria; a decrease in PTH concentration of > 50% was seen in 11 patients and 8 patients had PTH levels that fell to within the NR. The clinical characteristics of the patients according the persistence of disease are summarized in *Table 1*.

The results of univariate analysis comparing patients in whom treatment was successful with those who experienced disease persistence are presented in *Table 1*.

The analysis showed a statistically significant difference for both tests examined (*P* < 0.001). The presence of double adenoma or hyperplasia on histology, and thus the presence of multiglandular disease (*P* = 0.001), was a significant predictor of the persistence of hyperparathyroidism. Concurrent thyroidectomy increased the risk of recurrence.

Multivariate analysis confirmed the efficacy of using both ioPTH criteria to reduce persistence (*Table 2*). Further, multiglandular disease, specifically double adenoma or hyperplasia, was associated with a higher risk of disease persistence after surgery. In seven patients, the surgery did not result in the removal of any pathological glands, despite the surgeon performing BNE because there was no decrease in ioPTH concentration . Two of these seven patients did not experienced disease recurrence within 6 months after surgery, and are still being followed up. The remaining five patients exhibited disease persistence. In the multivariate analysis, these seven patients were treated as a distinct category to avoid their forced inclusion into either the multiglandular or uniglandular pathology (adenoma and carcinoma) groups. This approach was adopted to preserve the integrity of the statistical results, because including these patients in the predefined categories could have either artificially strengthened or weakened the overall analysis. Maintaining them as a separate category ensured that the analysis remained unbiased, and that no potential distortions were introduced as a result of misclassifying these patients. This decision was also validated by a sensitivity analysis, which confirmed that keeping these patients in the analysis did not compromise the robustness of the results.

**Table 2:** Multivariate logistic regression analysis of variables associated with persistence of primary hyperparathyroidism

	Odds ratio	z	*P*
**ioPTH**			
Fall to within normal range	0.12 (0.03, 0.53)	−2.84	0.005
Reduction > 50%	0.21 (0.05, 0.92)	−2.07	0.039
**Histological findings**			
Adenoma and carcinoma	1.00 (reference)		
Hyperplasia	10.53 (2.26, 48.99)	3.00	0.003
Inconclusive	205.27 (19.38, 2173.98)	4.42	< 0.001
MEN1	5.42 (0.32, 91.09)	1.17	0.240
Preoperative serum calcium	0.70 (0.45, 1.09)	−1.57	0.120
Surgical approach (MIP *versus* open surgery)	0.15 (0.02, 1.04)	−1.92	0.050
Concurrent thyroidectomy	5.33 (0.74, 38.43)	1.66	0.097

Values in parentheses are 95% confidence intervals. Variables were included in the model based on the results of univariate analysis (*P* < 0.250) and on expert opinion. ioPTH, intraoperative parathyroid hormone; MEN1, multiple endocrine neoplasia type 1; MIP, minimally invasive parathyroidectomy.

A reduction of > 50% in PTH concentration 10 min after glandular excision showed higher sensitivity than ioPTH values falling to within the NR (95.0 *versus* 81.1%, respectively), enabling better detection of cured patients (*Tables 3* and *4*). Using this criterion prevented surgeons from performing unnecessary neck dissections. Conversely, the ioPTH level falling to within the NR had higher specificity than the > 50% reduction in PTH (60.0 *versus* 45.0%, respectively), enabling the identification of patients with persistent disease who require BNE (*Tables 3* and *4*). However, using this criterion may lead to unnecessary BNE, despite a lower number of patients with persistent disease after surgery.

**Table 3:** Utility of a > 50% reduction in intraoperative parathyroid hormone level (Miami criterion) for predicting surgical outcome

Sensitivity (%)	Specificity (%)	PPV (%)	NPV (%)	Accuracy (%)
95.0 (92.2, 97.0)	45.0 (23.1, 68.5)	96.9 (94.5, 98.4)	33.3 (16.5, 54.0)	92.4 (89.2, 94.8)

Values in parentheses are 95% confidence intervals, calculated using the Clopper–Pearson method. PPV, positive predictive value; NPV, negative predictive value.

**Table 4:** Utility of a fall in intraoperative parathyroid hormone level to within the normal range for predicting surgical outcome

Sensitivity (%)	Specificity (%)	PPV (%)	NPV (%)	Accuracy (%)
81.1 (76.7, 85.0)	60.0 (36.1, 80.9)	97.3 (94.8, 98.8)	15.0 (8.0, 24.7)	80.0 (75.6, 83.9)

Values in parentheses are 95% confidence intervals, calculated using the Clopper–Pearson method. PPV, positive predictive value; NPV, negative predictive value.

The combination of both ioPTH criteria exhibited higher sensitivity than either test alone, with a specificity between that of each of the two tests and the highest accuracy (*Table 5*). By combining these ioPTH criteria, surgeons can enhance their ability to detect patients who have been treated successfully, thus avoiding unnecessary invasive procedures and consequently reducing the persistence rate even more effectively than simply relying on a decrease in ioPTH level of > 50% alone.

**Table 5:** Utility of combining a drop in intraoperative parathyroid hormone level to within the normal range with the Miami criterion for predicting surgical outcome

Sensitivity (%)	Specificity (%)	PPV (%)	NPV (%)	Accuracy (%)
97.5 (95.3, 98.9)	55.0 (31.5, 76.9)	97.5 (95.3, 98.9)	55.0 (31.5, 76.9)	95.3 (92.6, 97.2)

Values in parentheses are 95% confidence intervals, calculated using the Clopper–Pearson method. PPV, positive predictive value; NPV, negative predictive value.

## Discussion

The introduction of rapid ioPTH monitoring has enabled intraoperative prediction of surgical success, reducing the risk of persistence^10–13,17^. Often it is not possible to determine the number of parathyroid glands involved, their exact location, or the underlying pathology at the time of surgery^4,18,19^. Given that reoperation for persistent or recurrent disease is particularly complex because of scar tissue and carries a high risk of both intraoperative and postoperative complications, it is crucial to ensure that all pathological tissue is removed during the initial surgery^5,8,20^. The use of rapid ioPTH monitoring has also facilitated the emergence of MIP, which limits bilateral exploration and associated complications by focusing the intervention on the removal of the gland suspected on preoperative imaging^21,22^.

The results of the present study indicated that the decrease in ioPTH concentration to within the NR, although effective in contributing to the prevention of disease persistence due to its high specificity, may lead to an increased number of unnecessary bilateral explorations owing to lower sensitivity. Conversely, a decrease in ioPTH level of > 50%, although useful in minimizing unnecessary bilateral explorations due to its higher sensitivity, is associated with a higher risk of disease persistence as a result of its lower specificity. Using a combination of the two criteria yields the best results. Thus, when the decrease in ioPTH level at 10 min is < 50% but falls within the NR, the surgical intervention can be completed without an increased risk of persistence. In contrast, even when a decrease in ioPTH concentration to within the NR has not been achieved, a 50% decrease relative to baseline helps avoid unnecessary BNE^23,24^. The authors recommend the combined use of a decrease of > 50% in ioPTH level and a fall in ioPTH level to within the NR to achieve the best surgical outcomes. This strategy offers a balance between avoiding unnecessary bilateral explorations and ensuring a low persistence rate, optimizing the advantages of ioPTH monitoring.

One of the downsides of ioPTH measurement is prolonged operating time, both in terms of operating room occupancy and the duration of general anaesthesia for the patient. This is especially the case in centres where the analysis cannot be performed in the operating room owing to equipment costs and must be conducted at the hospital's central laboratory. Although ioPTH monitoring offers clear overall benefits in improving surgical outcomes for patients with primary hyperparathyroidism, it is important to balance these advantages against potential drawbacks, such as costs and the risk of false results. The effectiveness of ioPTH monitoring depends on the surgeon's expertise, and interpretation of the results^25,26^.

Among the widest adopted criteria is the Miami criterion, which requires a decrease of > 50% between the PTH level at baseline (or immediately before excision) and that 10 min after excision, and has an estimated accuracy of approximately 97%^12^. The most recognized criterion is the return of PTH levels to within the NR, known as the Halle criterion and first described by Riss *et al*. in 2007^27^. This criterion requires that PTH levels decrease within 15 min after gland excision to the ‘low’ normal range, set at 35 ng/l. With a specificity of 100%, this criterion accurately classifies all diseased patients, but may lead to a number of unnecessary BNEs, resulting in an accuracy of only 65%.

In their 2011 study, Richards *et al*.^15^ attempted to validate the association between the Miami criterion and the reduction in ioPTH to a ‘near-normal’ PTH level. However, the use of heterogeneous samples for analysis, an unclear and operator-dependent definition of near normal that varies depending on the patient, and the surgeon's discretion significantly weaken the reliability and applicability of the study’s findings to the clinical setting.

As demonstrated in the present study, both criteria have their advantages and limitations. The Miami criterion has higher sensitivity and a slightly better NPV, suggesting that it may be more reliable in correctly detecting surgical success, although at the cost of misclassifying untreated patients, thereby contributing to higher persistence rates. Conversely, the criterion of the ioPTH level returning to within the NR has slightly higher specificity, reducing the risk of false-positives and providing greater confidence in positive surgical outcomes. However, this criterion has a slightly lower sensitivity and a very low NPV, indicating an increased number of unnecessary BNEs.

The combination of both ioPTH criteria shows higher sensitivity (97.5%), indicating very good ability to correctly classify surgical success and identify patients who require BNE. The specificity (55.0%) of the combination is moderate; it is significantly better than the specificity of the criterion of a > 50% reduction in ioPTH level, but lower than that of return of the ioPTH concentration to within the NR. This indicates a higher risk of false-positives with the combination than with using the NR as a threshold. In this instance, the combination of both criteria reduces the number of patients with disease persistence compared with use of the > 50% reduction criterion alone, but does not reach the performance of the NR criterion, which remains the most reliable parameter for demonstrating patient cure. The combination has a significantly high PPV (97.5%), suggesting that, if the combination of criteria suggests surgical success, it is highly likely to be so. In addition, the combination has a significantly improved NPV (55.0%) compared with the two individual criteria, meaning that there is a moderate likelihood that a negative result actually reflects surgical failure. Therefore, the risk of performing bilateral explorations or continuing surgery unnecessarily is reduced compared with application of the two criteria individually. The use of this combination of criteria allows a reduction in BNEs and associated complications, ensuring an appropriate balance between the risk of persistence owing to the false-positive rate and optimization of the drawbacks related to use of the Miami criterion.

The discrepancies between the data reported in the literature^12,15,27^ and the findings of the present study may arise from several factors. First, although confidence intervals provide a more accurate assessment of a test's sensitivity and specificity, these intervals are often not reported, complicating comparisons. In addition, differences in the time limits used across various studies make direct comparisons difficult. Sample size also plays an important role, because smaller studies may show higher accuracy, which may not be valid in larger and more diverse populations. The present study included a larger and more representative patient population, with more complex disease, which may have partially reduced the performance of the two criteria but made the results more comparable with and applicable to general clinical practice.

This study presents an alternative interpretation of ioPTH measurement compared with the criteria most commonly adopted in clinical practice and those widely studied in literature. The results, obtained from a large sample size, using sophisticated statistical analysis, rigorous methods, and a representative patient population, provide insights into the use of ioPTH monitoring only partially addressed in previous studies.

Although this study provides valuable insights into ioPTH measurement, it is important to acknowledge several limitations inherent to the study. First, the inclusion of patients with MEN1 and patients with non-diagnostic or absent pathological findings may have introduced variability in the results. Second, there may have been changes in clinical practice or diagnostic techniques over the long inclusion period of 20 years, potentially influencing the study's outcomes. Finally, the study relies on retrospective data from a prospectively maintained database, which, despite its strengths, is subject to biases and constraints typical of retrospective analyses.

## Supplementary Material

zraf055_Supplementary_Data

## Data Availability

Data are not available owing to privacy and ethics restrictions.
