# Supplementary material for: Investigating intraoperative parathyroid hormone criteria for enhanced accuracy and surgical success in treating primary hyperparathyroidism: results from two tertiary referral centres
Source: BJS Open. 2025 Jun 13;9(3):zraf055. doi: 10.1093/bjsopen/zraf055 (PMC12163589; doi:10.1093/bjsopen/zraf055)
Supplement: zraf055_Supplementary_Data [file zraf055_supplementary_data.doc]

STROBE Statement—Checklist of items that should be included in reports of ***case-control studies***

The order and presentation format have been adapted according to our preferences, the journal's style, and the traditions of our specific research field.

|  | Item No | Recommendation |
| --- | --- | --- |
| **Title and abstract**  **p. 1-2** | 1 | (*a*) Indicate the study’s design with a commonly used term in the title or the abstract  *Title p. 1 and Abstract p. 2.* |
|  |  | (*b*) Provide in the abstract an informative and balanced summary of what was done and what was found  *Abstract p. 2 (line 39-59)* |
| Introduction p. 3-4 | | |
| Background/rationale | 2 | Explain the scientific background and rationale for the investigation being reported  *p. 3-4 (lines: 64-92)* |
| Objectives | 3 | State specific objectives, including any prespecified hypotheses  *p. 4 (lines 93-96)* |
| Methods p. 4-6 | | |
| Study design | 4 | Present key elements of study design early in the paper  *p. 4-5* |
| Setting | 5 | Describe the setting, locations, and relevant dates, including periods of recruitment, exposure, follow-up, and data collection  *p. 4 (lines 100-115)* |
| Participants | 6 | (*a*) Give the eligibility criteria, and the sources and methods of case ascertainment and control selection. Give the rationale for the choice of cases and controls  *p. 4-5 (lines 110-122)* |
|  |  | (*b*) For matched studies, give matching criteria and the number of controls per case |
| Variables | 7 | Clearly define all outcomes, exposures, predictors, potential confounders, and effect modifiers. Give diagnostic criteria, if applicable  *p. 5-6 (lines 110-145)* |
| Data sources/ measurement | 8* | For each variable of interest, give sources of data and details of methods of assessment (measurement). Describe comparability of assessment methods if there is more than one group  *p. 6 (lines 138-145)* |
| Bias | 9 | Describe any efforts to address potential sources of bias  *Retrospective study* |
| Study size | 10 | Explain how the study size was arrived at 380 patients  *Retrospective study and Fig.1* |
| Quantitative variables | 11 | Explain how quantitative variables were handled in the analyses. If applicable, describe which groupings were chosen and why  *p. 5-6* |
| Statistical methods  *p. 6* | 12 | (*a*) Describe all statistical methods, including those used to control for confounding |
|  |  | (*b*) Describe any methods used to examine subgroups and interactions |
|  |  | (*c*) Explain how missing data were addressed |
|  |  | (*d*) If applicable, explain how matching of cases and controls was addressed |
|  |  | (*e*) Describe any sensitivity analyses |
| Results p. 6-9 | | |
| Participants | 13* | (a) Report numbers of individuals at each stage of study—eg numbers potentially eligible, examined for eligibility, confirmed eligible, included in the study, completing follow-up, and analysed  *p. 6 and tab. 1* |
|  |  | (b) Give reasons for non-participation at each stage  *Retrospective study, only patients with 6 months of follow-up were included* |
|  |  | (c) Consider use of a flow diagram  *Fig. 1* |
| Descriptive data | 14* | (a) Give characteristics of study participants (eg demographic, clinical, social) and information on exposures and potential confounders  *p. 6-7 Tab 1* |
|  |  | (b) Indicate number of participants with missing data for each variable of interest  *Retrospective study, patients with missing data were excluded* |
| Outcome data | 15* | Report numbers in each exposure category, or summary measures of exposure  *p. 6-7 and Tab1.* |
| Main results  *p. 6-9* | 16 | (*a*) Give unadjusted estimates and, if applicable, confounder-adjusted estimates and their precision (eg, 95% confidence interval). Make clear which confounders were adjusted for and why they were included. |
|  |  | (*b*) Report category boundaries when continuous variables were categorized |
|  |  | (*c*) If relevant, consider translating estimates of relative risk into absolute risk for a meaningful time period. |

| Other analyses | 17 | Report other analyses done—eg analyses of subgroups and interactions, and sensitivity analyses  *p.8 (lines 205-212)* |
| --- | --- | --- |
| Discussion p.9-12 | | |
| Key results | 18 | Summarise key results with reference to study objectives  *p.9-11 (lines 234-285)* |
| Limitations | 19 | Discuss limitations of the study, taking into account sources of potential bias or imprecision. Discuss both direction and magnitude of any potential bias  *p. 11 (lines 297-303)* |
| Interpretation | 20 | Give a cautious overall interpretation of results considering objectives, limitations, multiplicity of analyses, results from similar studies, and other relevant evidence  *p.9-11 (lines 234-285)* |
| Generalisability | 21 | Discuss the generalisability (external validity) of the study results  *p.9 (lines 234-245)* |
| Other information | | |
| Funding | 22 | Give the source of funding and the role of the funders for the present study and, if applicable, for the original study on which the present article is based.  *No founding* |

*Give information separately for cases and controls.

**Note:** An Explanation and Elaboration article discusses each checklist item and gives methodological background and published examples of transparent reporting. The STROBE checklist is best used in conjunction with this article (freely available on the Web sites of PLoS Medicine at http://www.plosmedicine.org/, Annals of Internal Medicine at http://www.annals.org/, and Epidemiology at http://www.epidem.com/). Information on the STROBE Initiative is available at http://www.strobe-statement.org.
